# Left atrial functional remodeling following percutaneous closure of atrial septal defect secundum in adult patients

**DOI:** 10.1186/s43044-025-00700-9

**Published:** 2025-11-13

**Authors:** Pramadya Vardhani Mustafiza, Lucia Kris Dinarti, Real Kusumanjaya Marsam, Hasanah Mumpuni, Dyah Wulan Anggrahini

**Affiliations:** 1 Sebelas Maret University, Surakarta, Indonesia; 2 Dr Moewardi General Hospital, Surakarta, Indonesia; 3 Gadjah Mada University, Yogyakarta, Indonesia; 4 Rumah Sakit Umum Pusat Dr. Sardjito, Yogyakarta, Indonesia

**Keywords:** Atrial septal defect secundum, Left atrial strain, Percutaneous device closure, Speckle tracking echocardiography

## Abstract

**Background:**

Atrial septal defect secundum (ASDs) is one of the congenital heart diseases that is common in adults and frequently associated with volume overload and functional impairment of the right heart. Percutaneous device closure has become the preferred treatment because of its efficacy and lower complication rates. While right heart remodeling post-closure is well established, the effects on left atrial (LA) function, especially phasic components involving reservoir, conduit, and contraction strain, remain less understood. Two-dimensional speckle tracking echocardiography (2D STE) enables early and sensitive detection of LA functional changes.

**Method:**

This retrospective cohort study included adult patients (aged 18–65 years) with ASDs who underwent percutaneous device closure between December 2022 and October 2023 at Dr. Sardjito Hospital, Yogyakarta, Indonesia. Echocardiographic evaluations, including LA strain analysis (LASr, LAScd, LASc), were performed pre-closure, within 6 months (1st semester), and between ≥ 6–12 months (2nd semester) post-device closure. Patients with significant valvular disease, persistent arrhythmias, or suboptimal acoustic windows were excluded. Statistical analysis was conducted using repeated measures ANOVA or Friedman tests with Bonferroni correction.

**Result:**

A total of 21 patients (mean age 40.86 ± 14.74 years; 76% female) were included. LA reservoir strain (LASr) significantly declined in the 1st semester post-device closure compared to baseline (*p* = 0.003) and showed partial recovery by the 2nd semester (*p* = 0.016 vs. baseline). LA conduit strain (LAScd) and contraction strain (LASc) also significantly decreased in the 1st semester (*p* = 0.030 and *p* = 0.025, respectively), while LASc did not significantly improve at the 2nd semester follow-up. No significant changes were observed in LA geometry. Interobserver agreement for all strain measurements was excellent (ICC ≥ 0.99).

**Conclusion:**

LA functional impairment was observed in the 1st semester after percutaneous device closure, marked by a noticeable decline in all strains of the LA (LASr, LAScd, and LASc). Partial recovery of LASr was observed at one year, whereas LASc did not show significant improvement after the initial reduction. These findings highlight that the LA strain may be a sensitive marker for predicting the development of LA dysfunction post-device closure follow-up.

## Background

Atrial septal defect secundum (ASDs) accounts for about 80% of all atrial septal defect (ASD) types, typically occurring in the mid-atrial septum [1]. In its early phase, a left-to-right shunt leads to right heart volume overload, and over time, it may cause atrial arrhythmias, reduced exercise tolerance, and pulmonary hypertension [2, 3]. Over the last decade, percutaneous device closure has become the preferred treatment over surgery [4] due to its comparable efficacy, minimal complications, and proven safety in both short- and long-term follow-up [5, 6].

Chronic RV volume overload is a hallmark of ASD’s natural history, which eventually results in right chamber enlargement, dysfunction, and chronic heart failure [7]. Percutaneous ASD closure eliminates the left-to-right shunt, reduces RV volume overload, and promotes rapid reverse remodeling, with improvements seen as early as the day after the procedure and sustained over time. Closure of ASD thereby improves right heart chamber anatomy, geometry, and function in addition to symptoms [8–11].

On the other side, sudden shunt reduction in closure of ASDs can initially increase left atrial (LA) pressure. While right heart volume overload improves within six months, abrupt RV unloading can increase LA stiffness from the first day post-closure, and becomes more pronounced six months later. This correlation between RV remodeling and altered LA functional properties highlights the need for thorough LA function assessment after ASD closure [12–16]. Currently, there are few studies that examine changes in LA function after ASD closure.

Strain, also known as myocardial deformation, was a dimensionless measure that describes the change in myocardial size or shape in response to applied mechanical forces. It reflected local myocardial function and could be obtained using myocardial Doppler echocardiography measurement [17]. LA strain was functionally divided into three distinct phases: reservoir, conduit, and contraction [18, 19]. Previous studies have shown that the LA strain provides earlier and more sensitive early detection of atrial dysfunction than other geometric methods [20, 21].

Global and regional LA function can be assessed using strain and strain rate as determined by two-dimensional speckle-tracking echocardiography (2D STE). Phasic LA function consists of left atrial reservoir strain (LASr), left atrial conduit strain (LAScd), and left atrial contraction strain (LASc). LA filling is accomplished during the reservoir phase, which lasts from the end of ventricular diastole to the opening of the mitral valve. The conduit phase lasts from the opening of the mitral valve to the start of the LA contraction in early diastole. From the beginning of the LA contraction until the mitral valve closes in sinus rhythm, the contractile phase takes place during late diastole. Preload and LA relaxation can alter reservoir function. LV relaxation and LA afterload can have an impact on conduit function. Intrinsic LA contraction can alter contractile function [22].

The aim of this study was to assess the impact of percutaneous ASDs closure on LA function using 2D STE in adult patients.

## Methods

### Data collections

This study design is an observational retrospective cohort. We collected patients aged 18–65 years, with ASDs suitable for device closure, who underwent transcatheter closure between December 2022 and October 2023 at Dr. Sardjito Hospital, Yogyakarta, Indonesia. Follow-up was carried out for 1 year with echocardiography evaluation at least twice, the first in the 1st semester after procedure (< 6 months) and then in the 2nd semester after closure (≥ 6–12 months). We collected pre-ASDs closure data including age and gender. We excluded patients who had arrhythmia at baseline or developed arrhythmia throughout the one-year follow-up period. In our study, we excluded patients who had atrial fibrillation at baseline, but there was no development of arrhythmia during the follow-up period. Other exclusion criteria include patients with more than mild mitral regurgitation, and significant left ventricular dysfunction, and other persistent arrhythmias, as well as those in whom LA strain measurements could not be obtained due to technical issues (e.g., device problems, suboptimal acoustic windows), were excluded from the study.

### Echocardiographic assessment

Echocardiographic assessments were conducted using GE Vivid E95. Parasternal long-axis and apical two- and four-chamber views were acquired with an M5S probe operating at 2–4 MHz. Transthoracic echocardiography was performed at three time points: before closure, in the 1st semester after closure (< 6 months), and in the 2nd semester post-device closure (≥ 6–12 months).

The collected echocardiographic parameters included left ventricular systolic function, measured for left ventricular end diastolic diameter (LVEDd), left ventricular end systolic diameter (LVEDs), interventricular septal thickness at end diastole (IVSd), interventricular septal thickness at end systole (IVSs), relative wall thickness (RWT), left ventricular mass index (LVMI), and ejection fraction (EF); left ventricular diastolic study measured for early to late diastolic trans-mitral flow velocity (E/A), early mitral inflow velocity (E), velocity of the lateral E-wave (e’ lateral), velocity of the septal E-wave (e’ septal), early mitral inflow velocity and mitral annular early diastolic velocity ratio( E/e’ ); and left atrial study measured for LA diameter, left atrial volume index (LAVI), LA strain function (reservoir, conduit, and contraction) using 2D STE.

LA strain curves were obtained using R–R interval gating. To ensure optimal analysis, images were acquired with a high frame rate (50–90 Hz) and adequate imaging depth, focusing specifically on the LA to prevent foreshortening. The region of interest (ROI) for the LA was manually adjusted for each image, carefully excluding adjacent structures, including the pericardium, pulmonary veins, and LA appendage. From the calculated strain curve, values of LASr, LAScd, and LASc were obtained.

The 2D strain rate of LA was measured with speckle tracking echocardiography in apical four-chamber view, performed with EchoPAC software for GE Vivid E95. Echocardiography examinations were performed by certified cardiovascular echocardiography technicians or cardiac sonographers, analyzed by two specialists in cardiac imaging echocardiography, and then the interobserver variability was assessed. Interobserver variability, expressed as a coefficient of variation, was assessed by analyzing all segments, randomly chosen subjects, and blinded to patient information and time points from two independent investigators. Statistical analysis of the data was conducted by one experienced investigator.

### Statistical analysis

Statistical analyses were conducted using the IBM SPSS 25 software package. The median (interquartile range) or mean ± standard deviation was used to display continuous variables. The Shapiro-Wilk test was used to determine the normal distribution of continuous variables. To find any statistically significant changes across follow-up times, repeated ANOVA measures were conducted to see if the data were normally distributed. The Bonferroni adjusted multiple comparison test was used to identify the follow-up times that contributed to any statistically significant differences found by the variance analysis. The Friedman test was used to identify any significant differences in the median values across follow-up times in cases where the data is not normally distributed. The Bonferroni corrected Wilcoxon Signed Rank test was used to identify the follow-up times that resulted in a statistically significant difference in the Friedman test. The intraclass correlation coefficient (ICC) with 95% limits of agreement (LOA) was used to assess interobserver variability. For every analysis, a p-value of less than 0.05 was deemed to indicate statistical significance.

## Results

From December 2022 to October 2023, we treated 38 patients with ASDs. There were 21 patients in total after the data were adjusted to remove those who did not fit the exclusion criteria (Table 1).

**Table 1:** Baseline characteristics of patients

Characteristics (*n* = 21)	Mean ± SD
Age	40.86 ± 14.74
Sex	16 (76,19%) females 5 (23.81%) males
LA function	
LA diameter (mm)	34.95 ± 5.86
LAVI (ml/m^2^)	32.83 ± 14.89
LASr(%)	33.78 ± 14.80
LAScd (%)	21.17 ± 10.67
LASc (%)	12.61 ± 7.96
LV systolic function	
LVIDd (mm)	36.78 ± 4.62
LVIDs (mm)	21.90 ± 3.07
IVSd (mm)	7.78 ± 1.29
IVSs (mm)	10.94 ± 2.16
RWT	0.44 ± 0.10
LVMI (g/m²)	51.43 ± 11.11
EF (%)	71.78 ± 6.40
LV diastolic function	
E/A	1.25 ± 0.28
E (cm/s)	82.75 ± 28.76
e’lat (cm/s)	15.04 ± 3.89
e’ med (cm/s)	10.17 ± 3.22
E/e’	6.75 ± 2.38

Table 2 shows the changes in baseline characteristics of echocardiographic parameters before closure, after the 1st semester post-device closure, and after the 2nd semester post-device closure. Significant differences were obtained in the LASr group (p = 0.002), LVIDd (p < 0.001), LVIDs (p < 0.001), IVSs (p = 0.015), LVMI (p < 0.001), E/A (p = 0.047), and E/e’ (*p* = 0.037), but there were no significant differences in other parameters.

LASr showed a noticeable decline in the 1st semester, followed by a partial recovery in the 2nd semester (overall *p* = 0.002). Similarly, LASc showed a decline in the 1st semester, with a trend towards recovery in the 2nd semester (overall *p* = 0.127). On the other hand, the LAScd trend showed a decrease after the 1st semester and further decrease at the 2nd semester compared to the baseline with no recovery (*p* = 0.030 and *p* = 0.058, respectively) (Table 2).

**Table 2:** Changes in echocardiographic parameters before and after device closure

Characteristics	Pre closure (*n* = 21)	1st semester post-device closure (*n* = 21)	2nd semester post-device closure (*n* = 21)	*p*
	Mean ± SD	Mean ± SD	Mean ± SD	
LA function				
LA diameter (mm)	34.95 ± 5.86	37.00 ± 4.87	36.24 ± 6.27	0.096
LAVI (ml/m^2^)	32.83 ± 14.89	31.73 ± 17.39	34.35 ± 13.30	0.405
LASr(%)	33.78 ± 14.80	23.93 ± 10.76	26.46 ± 11.62	0.002*
LAScd (%)	21.17 ± 10.67	17.00 ± 8.07	16.32 ± 8.98	0.172
LASc (%)	12.61 ± 7.96	7.02 ± 5.12	10.18 ± 3.77	0.127
LV systolic function				
LVIDd (mm)	36.78 ± 4.62	40.05 ± 4.54	45.65 ± 3.71	< 0.001*
LVIDs (mm)	21.90 ± 3.07	23.80 ± 3.04	26.88 ± 4.06	< 0.001*
IVSd (mm)	7.78 ± 1.29	8.95 ± 2.42	8.65 ± 1.81	0.078
IVSs (mm)	10.94 ± 2.16	12.65 ± 2.47	11.53 ± 2.35	0.015*
RWT	0.44 ± 0.10	0.43 ± 0.10	0.39 ± 0.08	0.181
LVMI (g/m²)	51.43 ± 11.11	68.51 ± 22.61	82.13 ± 17.99	< 0.001*
EF (%)	71.78 ± 6.40	71.45 ± 6.74	72.53 ± 6.57	0.800
LV diastolic function				
E/A	1.25 ± 0.28	1.59 ± 0.68	1.15 ± 0.45	0.047*
E (cm/s)	82.75 ± 28.76	89.76 ± 28.62	86.71 ± 20.09	0.598
e’lat (cm/s)	15.04 ± 3.89	14.17 ± 4.10	12.86 ± 3.65	0.077
e’ med (cm/s)	10.17 ± 3.22	8.92 ± 2.64	8.86 ± 2.41	0.066
E/e’	6.75 ± 2.38	8.53 ± 6.44	8.49 ± 2.85	0.037*

*LA*  left atrium, *LAVI*  left atrial volume index, *LASr*  left atrial reservoir strain, *LAScd*  left atrial conduit strain, *LASc*  left atrial contraction strain, *LVEDd*  left ventricular end diastolic diameter, *LVEDs*  left ventricular end systolic diameter, *IVSd*  interventricular septal thickness at end diastole, *IVSs*  interventricular septal thickness at end systole, *RWT*  relative wall thickness, *LVMI*  left ventricular mass index), *EF*  ejection fraction, *E/A*  early to late diastolic transmitral flow velocity, *E*  early mitral inflow velocity), *e’* lat velocity of the lateral E-wave, *e’ sep*  velocity of the septal E-wave, *E/e’* early mitral inflow velocity and mitral annular early diastolic velocity ratio. *=significant

LASr in Table 3 shows there was a significant difference between pre- and 1st semester post- (*p* = 0.003), 2nd semester post-device closure (*p* = 0.016) groups. In addition, LAScd and LASc in Table 3 also shows a significant difference between pre- and post-device closure, 1st semester (*p* = 0.030; *p* = 0.025) only. However, there were no significant differences between LASr, LAScd, and LASc values between the 1st semester post- and the 2nd semester post-device closure (Table 3).

**Table 3:** Comparison of LA strain groups between times

	Pre closure vs. 1st semester post device closure	Pre closure vs. 2nd semester post device closure	1st semester post device closure vs. 2nd semester post device closure
LASr	p = 0.003*	p = 0.016*	p = 0.305
LAScd	p = 0.030*	p = 0.058	p = 0.821
LASc	p = 0.025*	p = 0.414	p = 0.052

*LASr*  left atrial reservoir strain, *LAScd*   left atrial conduit strain, *LASc* left atrial contraction strain. *=significant

**Fig. 1:**
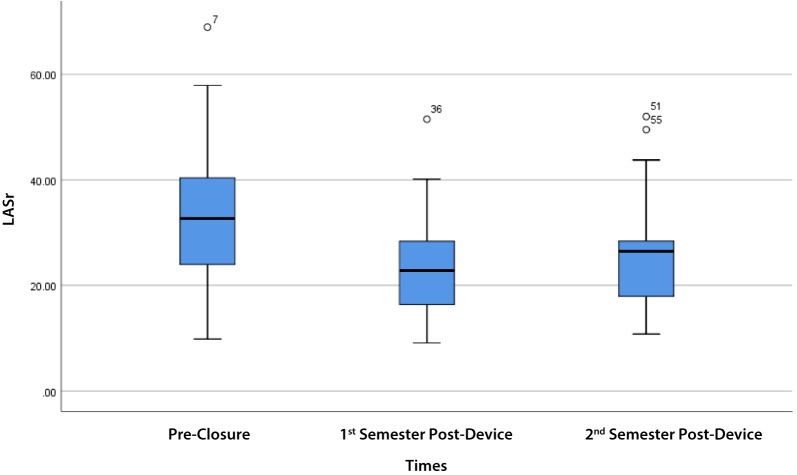
LASr trend pre-closure, 1st semester, and 2nd semester post-device closure

LASr significantly decreased from pre-closure to the 1st semester (*p* = 0.003) and remained significantly lower than pre-closure at the 2nd semester (*p* = 0.016). There was no significant difference in LASr between the 1st and 2nd semesters (*p* = 0.305), indicating a partial but not complete recovery to baseline (Fig. 1; Table 3).

**Fig. 2:**
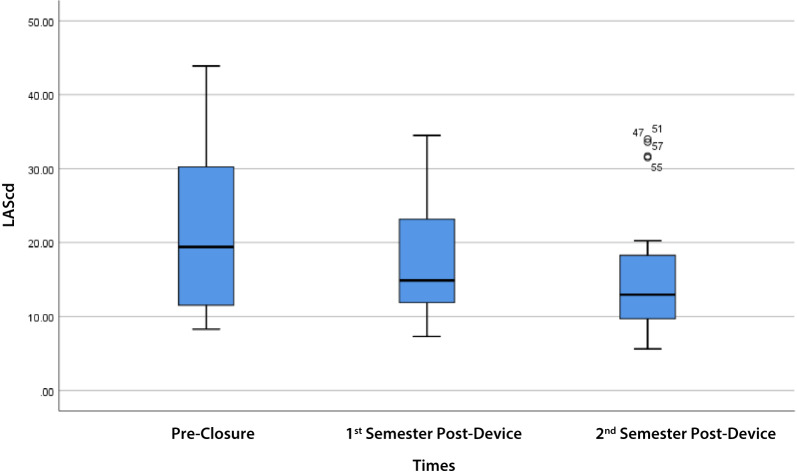
LAScd trend pre-closure, 1st semester, and 2nd semester post-device closure

LAScd values decreased significantly from pre-closure to the 1st semester (*p* = 0.030), but there was no significant difference to the 2nd semester (*p* = 0.058). There was no significant difference in the decreased value of LAScd between the 1st and 2nd semesters (*p* = 0.821), suggesting a lack of recovery to baseline (Fig. 2; Table 3).

**Fig. 3:**
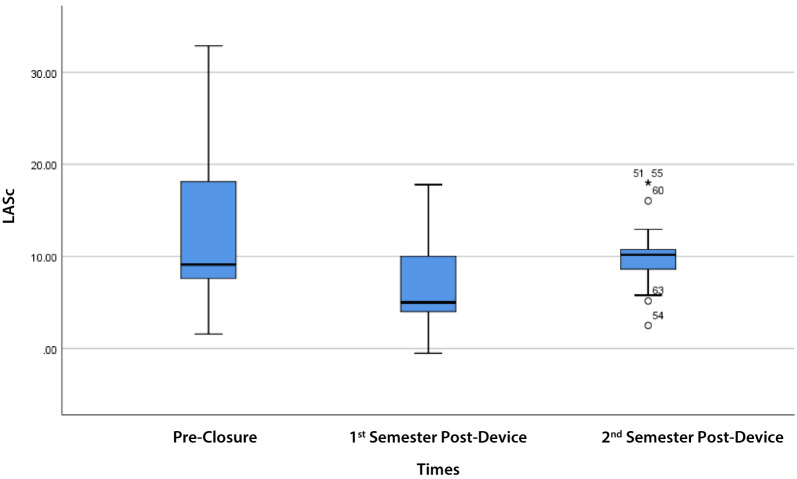
LASc trend pre-closure, 1st semester, and 2nd semester post-device closure

LASc values decreased significantly from pre-closure to the 1st semester (*p* = 0.025), but there was no significant difference in LASc from pre-closure at the 2nd semester (*p* = 0.414). There was no significant difference in LASc between the 1st and 2nd semesters (*p* = 0.052), indicating a partial but not complete recovery to baseline (Fig. 3; Table 3).

Global LA reservoir, conduit, and contractile strain interobserver correlations were 1.00 (95% LOA, 0.999-1.000; *p* < 0.001), 1.00 (95% LOA, 0.999-1.000; *p* < 0.001), and 0.99 (95% LOA, 0.998–0.999; *p* < 0.001).

## Discussion

Our study showed LA functional changes after percutaneous device closure without any significant LA geometrical changes. Significant decrease in LASr, LAScd, and LASc was obtained during 1st semester after percutaneous ASDs closure. When it comes to the 2nd semester follow-up, LASr and LASc have partial recovery compared to baseline. Reduction of LASr in the 2nd semester was not significant compared to the 1st semester, but it was still significant when compared to pre-closure. Our result was not in line with Thilen’s study and Pascotto’s study, which conclude remodeling LA after ASD closure is most pronounced immediately after treatment and terminates within 6 months following closure [23, 24]. There are several reports about the change of LA function after ASD closure, and the results were controversial.

In this study, LASr showed an initial significant decrease followed by partial recovery, though remaining below baseline at one year. A study from Suzuki et al. showed that LASr value decreased at 1 day after evaluation in the ASD-D (ASD closure with device) group with MD = − 14.43 (95% CI = − 17.88 to − 10.99; *p* < 0.0001) [25]. The similarity between these studies is that both show an initial drop of LASr post device-closure. A study from Choi et al. showed that the mean global LASr level decreased after six-months follow-up, with a value of before ASD closure 1.17 ± 0.34 and 0.85 ± 0.30 after ASD closure (*p* = 0.030) [22]. It showed no recovery after six months of follow-up. In contrast to this study, this research shows a decrease in LASr followed by partial recovery, while Choi et al.’s study [22] also decreased but showed no recovery. Aslan et al. [26] also evaluated LA function after ASD closure using volumetric measurement. They evaluated the LA function of 41 patients before closure, 1 day, and 6 months after closure. When comparing reservoir function one and six months after treatment to LA function prior to the operation, they found no difference [26]. This study shows a significant decrease in LASr, whereas Aslan et al.’s study [26] showed no change in LASr on the first day and sixth month compared with the baseline.

This study showed the LAScd value decreased after the 1st and 2nd semester compared to the pre-closure (*p* = 0.030 and *p* = 0.058, respectively). Study from Suzuki et al. [25] showed that LAScd function decreased 1 day after evaluation in the ASD-D group, and Choi et al.’s study which showed that the median global LAScd level decreased at six-month follow-up, with a value of before ASD closure 0.68 (0.42–1.16) and 0.41 (0.16–0.79) after ASD closure (*p* = 0.030) [22]. This study, together with those studies, shows a decrease in LASCd after device closure for at least 6 months of follow-up. Decrease of LAScd reflects impaired atrium conduit function. The atrium conduit function during the early diastolic LV filling phase was compromised due to resistance to the device. On the other hand, the study by Aslan et al. [26] showed different results. They reported that conduit function was improved after device closure [26].

From this study, LASc showed a significant initial drop followed by partial recovery, though remaining below baseline at one-year follow-up. A study from Suzuki et al. showed that LASc value decreased at 1 day after evaluation in the ASD-D (ASD closure with device) group with MD = − 3.54 (95% CI = − 5.63 to − 1.44; *p* = 0.0020) [25]. Aslan et al.’s showed that the mean global LA contractile strain level was decreased on the first day and sixth month after the procedure compared with the baseline [26]. The similarity between these studies is that both show an initial drop of LASc post device-closure at least within a 6-months follow-up. A study from Choi et al. showed the mean worldwide LA contractile strain level at the six-month follow-up was significantly better than the pre-procedural strain level (0.33 ± 0.65 vs. − 0.43 ± 0.38, *p* = 0.006) [22]. These results were not in line with that study. The difference with this study is this study shows an initial drop first (within 6 months) followed by partial recovery, whereas Choi et al.’s study [22] shows a direct improvement within 6-months follow-up.

The reduction of LA function in this study could be related to some factors. First, it has been known that after ASD closure, sudden shunt reduction can lead increase in left atrial pressure. Second, some studies are also reporting an increase of atrial stiffness after ASD closure regardless of the closure methods. Third is related to the impact of the device itself. Those factors potentially affected the ability of the left atrium to stretch and contract during the cardiac cycle. Our study’s findings were consistent with Suzuki et al., who found that early post-procedure LA dysfunction was caused by a resistance in LA blood conduction and a device interaction with atrial bundle integrity [25].

Structural changes of LA obviously can affect its function, or even more, lead to electrophysiological disorder, which is responsible for the development of atrial arrhythmias. Recently, studies about changes in LA function after ASD closure have increased, albeit some studies have reported inconsistent results. Several underlying reasons for those discrepancies were the timing of the LA function evaluation and differences in methods used.

This study had several limitations. First, it is a single-center, retrospective study with a small number of patients, which diminishes the applicability of the findings. Additional larger and longer-term follow-up studies should be considered. Second, the follow-up period was only one year, therefore, this study design does not enable an accurate long-term prognosis. Third, we did not analyze other confounding factors that could affect LA strain, including coexisting hypertension or coronary artery disease.

## Conclusion

Our study revealed that adult patients undergoing percutaneous closure of ASDs have a significant impairment of LA function, particularly LASr, within one year of follow-up compared to pre-closure values. These findings highlight that the LA strain may be a sensitive marker for predicting the development of LA dysfunction after device closure follow-up. Therefore, it may help to optimize pre- and post-device closure treatment and predict the long-term impact of LA dysfunction, including arrhythmia events (e.g., atrial fibrillation) and persistent diastolic dysfunction.

## Data Availability

No datasets were generated or analysed during the current study.

## Abbreviations

ASD: Atrial septal defect
ASDs: Atrial septal defects secundum
ASD-D: ASD closure with device
ASO: Atrial septal occluder
EF: Ejection fraction
E/A: Early to late diastolic transmitral flow velocity
E: Early mitral inflow velocity
e’: Lateral Velocity of the Lateral E-wave
e’: Septal Velocity of the Septal E-wave
E/e’: Ratio of Early Mitral Inflow Velocity to Mitral Annular Early Diastolic Velocity
ICC: Intraclass correlation coefficient
IVSd: Interventricular septal thickness at end diastole
IVSs: Interventricular septal thickness at end systole
LA: Left atrium
LASr: Left atrial reservoir strain
LAScd: Left atrial conduit strain
LASc: Left atrial contraction strain
LAVI: Left atrial volume index
LOA: Limits of agreement
LV: Left ventricle
LVEDd: Left ventricular end diastolic diameter
LVEDs: Left ventricular end systolic diameter
LVMI: Left ventricular mass index
RWT: Relative wall thickness
RV: Right ventricle
STE: Speckle tracking echocardiography
